# Remarks on Relations of the Physical Forces to Disease

**Published:** 1870-05

**Authors:** F. Homer Blackman


ARTICLE XIV.

### REMARKS ON RELATIONS OF THE PHYSICAL FORCES TO DISEASE.

BY F. HOMER BLACKMAN.

Etiology is the most difficult of all branches of medical study to elucidate. In this department of medical science, more than in any other, speculations and theories have occupied the place of facts. "All scientific inquiry begins in the same manner with guesses;" various conjectures and hypothesis being made to explain observed phenomena. Men are not all equally skilled in making generalizations. It depends mainly on boldness of thought and fertility of invention, an original cast of intellect, an inquisitive and suggestive frame of mind, which sees and prizes that which others neglect, which abides by no rules, and is a law unto itself. The celebrated chemist, Faraday, has said: "the world little knows how many thoughts and theories, which have passed through the mind of a scientific investigator, have been crushed in silence and secrecy by his own adverse criticism." All theories and generalizations are of use in scientific investigations, and have, in many instances, been productive of great good. It is probable that much of the discrepancy of opinions existing concerning the action of drugs, and our want of faith in medication, in general, is due to different observers holding dissimilar theories, by which they interpret the various phenomena of physiology and pathology. What seems to be wanting in medical science, more than anything else, is a central idea of life. We will never be able to rightly apprehend disease until we are able, in lucid terms, to state what life is. Life has hitherto been looked upon as a resultant of ordinary chemical forces, of the inorganic world, modified by a peculiar force called vital. In the advancing light of modern science, this vital force has flown, leaving no trace behind of its existence; and in its place there comes a "physical basis of life," plainly suggesting that there is some one kind of matter which is common to all living beings, and that their endless diversities are bound together by a physical as well as an ideal unity; showing conclusively that there is a "community of faculty between the bright-colored lichen, which so nearly resembles a mere mineral incrustation of the bare rock on which it grows, and the painter, to whom it is instinct with beauty, or the botanist, whom it feeds with knowledge."

The difference in faculty, in all living organisms is in degree, and not in kind. Contractility is manifest under the influence of certain stimuli, as well in the lowest plant as in the highest animal. There is a well-founded hope that presently we shall have a theory of life, which shall not have its origin in the idle vagaries of the imagination, but in substantial phenomena, which we are capable of appreciating and understanding. Scientific research has pretty fully demonstrated, that a threefold unity exists common in all life, namely: "A union of power or faculty, a unity of form, and a unity of substantial composition." The essential constituents of the "physical basis of life" is carbonic dioxide, water, and ammonia.

Modern science asserts of this matter of life, that it is composed of ordinary matter, "differing only in the manner in which its atoms are aggregated." Under whatever disguise it takes refuge—whether in the fungus, or oak, worm, or man—the living protoplasm not only ultimately dies and is resolved into its mineral and lifeless constituents, but is always dying; and, strange as the paradox may seem, could not live unless it died. In fact, in living organisms more than elsewhere, we see exemplified the great law, elaborated by Herbert Spencer, of the continuous redistribution of matter and motion. Man, in his ignorance, is fond of multiplying causes; but science, as if conscious of nature's love of unity and order, is constantly demonstrating the simplicity of truth. All living bodies are so constituted, that whatever disturbs the normal equilibrium of the redistribution of matter and motion is the cause of disease, the disease being the perturbation caused by the disturbed equilibrium.

The profession have, for a long time, tacitly asserted the belief, in many ways, that the causes of disease are much more suttle than text-books have described. Some seem almost to have reached the conclusion here adopted, that disease is a disturbance in the normal equilibrium of the forces existing in the organism. Dr. Gillette, of Philadelphia, in a report upon the "relations which electricity sustains to the causes of disease," strenuously advocates the doctrine, that almost all diseases are satisfactorily accounted for by electric disturbances in the atmosphere. Mr. Craig, of England, is a zealous advocate of similar views. The relations of temperature have been, and are still, being studied with intense interest; showing that among the thoughtful men of the profession, a feeling has existed for a long time, that caloric sustains an important relation to disease. It has not been studied in the direction that we could have wished, but, nevertheless, some important items may be gleaned from observations already made. The thermometer only tells us the relative amount of sensible heat evolved in the organism, but does not tell us anything about that which may remain in the system, destroying chemical affinity, by driving atom from atom. This can only be determined by studying the other physical forces in connection with temperature. This, then, is our ideal method of studying disease. Everything taken into the system is in relation to it, either a plus or a minus force.

The following conclusions seem to be warranted by recent scientific research, namely: that motion, chemical affinity, heat, light, electricity, magnetism, and nerve force are convertible, each into the other, both qualitatively and quantitatively; that large quantities of these forces surround every form of matter; that these forces are particularly subject to change, in their relative proportions, especially when existing in relation to that form of matter known as gaseous; that these forces, as they exist in the body, are evolved during the processes of respiration, digestion, and assimilation; and that they sustain a certain quantitative and qualitative relation to each other, which relation cannot be disturbed without more or less disastrous effects being produced in the organism; that the relations sustained to each other are qualitatively the same in living as in organic bodies; that the stability of the relations differ much in different organisms, and, also, in the different stages of their development and growth; and, finally, that no system of etiology which does not recognize the influence of the so-called physical forces, singly and combined, in the production of disease, is worthy of serious attention. Motion, where we can trace its genesis, we find to preëxist as some other mode of force. All our voluntary acts are preceded by certain sensations of muscular tension. The force accumulated in a body by downward motion is just equal to the force expended in its elevation; conversely arrested motion produces, under different circumstances, heat, light, electricity, magnetism, and chemical force; and, lastly, motion may be reproduced by the forces which have emenated from motion. Chemical force, or affinity, in its manifestations, exhibits some remarkable and well-ascertained phenomena. It is always accompanied by the production, or by the annihilation, of heat. A given amount of chemical action will absorb or evolve a certain definite amount of heat. Chemical action and heat are mutually convertible. The same quantity of heat evolved during a certain chemical action will be required, in order to reverse it. As has already been stated, chemical force is but a mode of motion. Now, it happens that bodies may possess so much chemical force or motion, that chemical affinity between them is impossible, or they may possess too little motion for chemical action. When metalic mercury is heated, nearly to the boiling point, and in that state exposed for a lengthened period to the air it oxidizes, if it then be raised to a still higher temperature, the oxide is decomposed. Paladium oxidizes superficially, at a red heat; but if the temperature rises to whitëness, the oxide is reduced and oxygen is set free. The vapor of water is decomposed by white hot platinum.

Chemists do not satisfactorily explain these phenomena, yet it seems to us that the explanation follows as a natural sequence to the phenomena themselves, and is in accordance with the fact previously stated, that too much or too little motion in a body prevents chemical affinity. Pathologists do not appear to have noted these facts, or, if having noted them, have failed to make any record of them.

A certain class of chemical phenomena known as catalytic, or action by presence, seems to be in accordance with the same law. For instance, we may have a mixture of two gases, in which no chemical action will take place, if kept in the dark, or, at all events, very slowly; but if exposed to the light, or if a current of electricity be passed through them, they will unite instantly. The introduction of a piece of spongy platinum into a mixture of oxygen and hydrogen gases causes chemical action, in a few minutes; the platinum becomes, at the same time, intensely heated. In the former instance, a certain amount of motion was necessary, in order to raise the motion in the gases, to the point of chemical affinity. In the latter case, the platinum acted as a conductor of motion, thus reducing the motion in the gases to the required degree. Death from lightning appears to be produced in harmony with the same law. Thus an immense amount of motion accumulating, for the time being, in and about the body, prevents chemical action.

Two varieties of sunstroke are described by authors. They may be thus described:—In one, the direct rays of the sun upon the head induces cerebral congestion; in the other variety, excessive heat, often not under the immediate influence of the sun, effects the whole system with prostration, apparently from a blood change; the chemical operations of the economy being modified by heat, in a manner incompatible with the vitality of the blood. Both are similar in cause, and differ only in degree. They are explainable by the chemical law previously stated, which holds good for all chemical action, whether it be in the body or out. The excessive amount of heat in the body prevents chemical changes. In the first variety, the heat induces violent chemical action, indeed, the very highest that can be produced. In the second variety, the heat has reached the point where it begins to prevent chemical affinity. Arctic explorers meet death, frequently, in a manner directly the reverse of this. The temperature of the frozen regions of the north being very low, heat is absorbed into the atmosphere with great rapidity. All the motion in the body is absorbed into the atmosphere in the form of heat. Intellection and muscular action are interfered with, and, finally, they cease altogether. The individual sinks quietly down in the snow, desires to be let alone; neither threats, persuasions, the entreaties of friends, or a knowledge of his situation have any influence over him. This phenomena is also explainable by our general law of too much or too little motion checking chemical action. Animals exhale carbonic dioxide, and, at the same time, evolve heat. Plants inhale carbonic dioxide and exhale oxygen, with the absorption of heat. The motion evolved in the formation of carbonic dioxide, in the body, is expended in the production of all those activities for which animals are noted. In the plant, an equivalent amount of motion must be absorbed, in order that chemical action may ensue, and carbonic acid be decomposed. To accomplish this, a certain amount of motion is obtained from the sun; and, in this manner, plants become storehouses of motion, and it is through them that animals indirectly obtain life from the sun.

In regard to the amount of motion contained in them, plants and animals hold an intermediate relation between the inorganic elements, composing the earth on which we live, and that great ocean of active, restless, uneasy matter called atmosphere, which is about and above us. The sun is the source from whence all these ceaseless activities arise. It is at once the fountain of life and the source of death. The temperature of the human body varies, in health, but slightly, from 98 degrees, while the atmosphere varies in a range of nearly 200. For health, the temperature of the atmosphere should be from 20 to 30 degrees below that of the body, thus permitting ready radiation of the evolved heat.

The fluctuations in temperature, observed to occur in many diseases, are due, probably, to retarded chemical changes, thus: when the heat accumulated in the body has reached a certain point, chemical change is diminished, until, by radiation, the temperature is reduced, when the same phenomena recurs. By recognizing this fact, we may pretty certainly predict much concerning our patients.

Epidemic cholera furnishes another striking illustration of the manner in which disease may be produced, by the sudden obstruction of some one of the physical forces from the system. This fact has been noted by observers, both in England and on the Continent. The force whose equilibrium is disturbed in this disease is electricity. In a communication to the French Academy, M. Andrand, who had charge of a very powerful electric machine, writes:—"I have remarked that since the invasion of cholera, I have not been able to produce, on any occasion, the same effect. Before its occurrence, in ordinary weather, after one or two turns of the wheel, brilliant sparks, of five or six centimeters in length, were given out. During the months of April and May, the sparks were obtained with difficulty, and their variations accorded very nearly with the variations of the cholera. During the days of the 4th, 5th, and 6th of June, it was impossible to obtain anything but slight cracklings, without sparks. On the 7th of June, the machine remained quite dumb. This new decrease of the electric fluid perfectly accords with the renewed violence of the cholera, as is only too well known. For my own part, I was more alarmed than astonished; my conviction was complete. At last, on the morning of the 8th, some feeble sparks reappeared, and from that hour the intensity decreased. Towards evening, a storm announced at Paris that the electricity had reëntered to its domain: to my mind it was the cholera that disappeared with the cause that produced it." In some experiments in England, during the epidemic of 1849, it was found that a magnet which ordinarily carried two pounds and ten ounces, would, when the atmospheric indications were at their worst—the air being saturated with moisture—sustain only one pound and ten ounces; the degree of its attraction varying with, and being in inverse proportion to, the violence of the disease. These phenomena are all readily explained by the general law previously enunciated, and is what we should, *a priori*, have been led to expect. In this case, the avenue through which motion made its escape was the electric. Those cases of cholera which occur without any violent evacuant symptoms show the different chemical affinity, which may be brought about in the body, by the abstraction of motion, in the form of electricity.

The matter composing the animal economy is subject to the same laws as that of the inorganic world; hence we should expect that all those circumstances which exercise a perturbing influence upon chemical action, outside of the body, should have a disturbing influence upon those taking place in the body. It should be our first object in studying disease, after this method, first, to study chemical change and its disturbing influences, in inorganic matter, then to apply the same principles to living organisms; recollecting that they owe their differences, not to different forces acting in their production, but to the matter out of which they are made.

